# QFitlia (Fitusiran): redefining hemophilia treatment with RNAi therapy. A correspondence

**DOI:** 10.1097/MS9.0000000000004674

**Published:** 2026-01-06

**Authors:** Govinda Lohano, Gaaitri Lohano, Riya R. Lohana, Sohana Memon, Sunita Lohana, Hermann Yokolo

**Affiliations:** a Department of Internal Medicine, Liaquat University of Medical and Health Sciences (LUMHS), Jamshoro, Pakistan; b Department of Internal Medicine, Jinnah Sindh Medical University, Karachi, Pakistan; c Department of Research, Medical Research Circle (MedReC), Goma, DR Congo

**Keywords:** fitusiran, hemophilia treatment, QFitlia, RNAi therapy

*Dear Editor,*

Hemophilia is the most common serious X-linked inherited bleeding disorder, caused by inherited or spontaneous *de novo* mutation^[1,2]^. Hemophilia A and B are caused by deficiencies in clotting factors VIII and IX, respectively, which inhibit thrombin production and hemostasis^[1–3]^. Following mild injuries, patients frequently suffer from protracted bleeding and, in extreme situations, spontaneous hemorrhage into muscles, joints, or important organs. Males are the primary victims of hemophilia; however, some female carriers may also show symptoms^[1,2]^. Coagulation factor VIII (FVIII) is crucial in hemostasis. Once activated, it joins activated factor IX (FIXa) on anionic cell membranes to form the Intrinsic Xase complex, which activates Factor X (FX), a key rate-limiting step in clot formation^[3]^. Treatment response is quite variable because around 30% of patients with hemophilia acquire inhibitory antibodies that decrease the effectiveness of factor replacement^[4]^. While patients with high-responding inhibitors usually need bypassing medications, those with low-responding inhibitors may continue factor replacement^[4]^. Other difficulties with intravenous factor treatment include the need for regular dosage, inadequate venous access, and the possibility of inhibitor formation^[5]^. Furthermore, immunological tolerance induction is expensive, time-consuming, and not always effective, and the Bethesda test is not a reliable indicator of bleeding risk^[4]^. All of these issues highlight the need for more potent treatments that are more widely applicable and have a reduced treatment burden.

This article complies with the TITAN 2025 guidelines – repressing the reporting and use of AI^[6]^.

QFitlia (Fitusiran), a first-in-class small interfering RNA treatment for patients aged ≥12 years with hemophilia A or B, with or without inhibitors, was approved by the U.S. Food and Drug Administration on 28 March 2025^[6,7]^. Fitusiran improves thrombin production and restores hemostatic balance regardless of inhibitor status by lowering hepatic antithrombin levels, in contrast to factor replacement^[6–8]^. Predictable effectiveness and a decreased need for frequent intravenous infusions are two useful benefits of this innovative technique. In the ATLAS-INH phase 3 trial, prophylactic fitusiran was compared with on-demand bypassing agents in patients with hemophilia and inhibitors. Of 57 participants, 38 received fitusiran prophylaxis and 19 received bypassing agents. The mean annualized bleeding rate was 1.7 with fitusiran versus 18.1 with bypassing agents, representing a 90.8% reduction (*P* < 0.0001). Notably, none of the fitusiran-treated participants experienced spontaneous treated bleeds, compared with 5% in the on-demand group^[8]^. Similarly, in ATLAS-A/B (patients without inhibitors), fitusiran prophylaxis achieved profound bleeding reduction compared with on-demand factor replacement, demonstrating efficacy in both hemophilia A and B^[9]^. Long-term extension studies confirmed durable bleeding control, including perioperative settings, with improvement in patient-reported quality-of-life metrics^[10]^. Despite its promise, QFitlia carries important safety considerations. Reported adverse events include elevated liver enzymes, thromboembolic complications, gallbladder disease (sometimes requiring cholecystectomy), and rare cases of liver toxicity^[7,8,11]^. Routine liver function monitoring is recommended at baseline and monthly for at least 6 months after treatment initiation or dose escalation^[7]^. Mild adverse events include nasopharyngitis and infections^[7]^. In order to balance effectiveness and thrombotic risk, QFitlia is injected subcutaneously, initially every 2 months, with dosage customized using the FDA-approved INNOVANCE^®^ Antithrombin test (Siemens Healthcare)^[7]^. It comes in vial-and-syringe formulations (10 mg and 20 mg) and a prefilled pen (50 mg), offering versatile administration choices that enhance adherence^[7]^. Particularly for patients with inadequate venous access or few alternatives for home-based prophylaxis, the subcutaneous route, occasional dose, and steady pharmacodynamic profile lessen treatment burden and promote practical use^[10]^.

Fitusiran enhances conventional hemophilia therapy by lowering treatment burden, maintaining constant hemostatic protection, and improving joint health and functional results, according to clinical data^[8–10]^. Fitusiran prophylaxis provides more consistent bleeding control in surgical and perioperative settings by reducing the requirement for high-dose factor replacement or bypassing medications^[10]^. All of these results point to how RNAi therapies might revolutionize the treatment of hemophilia in both inhibitory and non-inhibitor populations. As a straightforward, subcutaneous, RNAi-based treatment that successfully treats a variety of hemophilia subtypes and inhibitor statuses, QFitlia (Fitusiran) is a significant advancement in hemophilia care. With the potential to improve patient quality of life globally, its innovative mechanism, long-lasting effectiveness, controllable safety profile, and decreased treatment burden represent a significant improvement over conventional factor replacement therapy^[6–10]^.

## Data Availability

Not applicable.
